# 

**Published:** 1972-01

**Authors:** 


					B^SrO
CH|feuL
NATIONAL LIBRARY OF MEDiC,NE \
P.SCQ. OCT 25 t972
-rn Wl
INDEX
INPUT nov aUK-i
JANUARY 1972
VOL. 87 (i) No. 321

				

